# The molecular subtypes and clinical prognosis characteristic of tertiary lymphoid structures-related gene of cutaneous melanoma

**DOI:** 10.1038/s41598-023-50327-6

**Published:** 2023-12-28

**Authors:** Juan Li, Gang Chen, Yang Luo, Jin Xu, Jun He

**Affiliations:** 1 Chongqing Academy of Chinese Materia Medica, Chongqing, People’s Republic of China; 2 Present Address: Chonging College of Traditional Chinese Medicine, Bishan District, 61 Puguoba Road, Bicheng Street, Chongqing, 402760 People’s Republic of China

**Keywords:** Cancer, Skin cancer, Melanoma

## Abstract

Despite the remarkable efficacy of PD-1-associated immune checkpoint inhibitors in treating cutaneous melanoma (CM), the inconsistency in the expression of PD-1 and its ligand PD-L1, and resulting variability in the effectiveness of immunotherapy, present significant challenges for clinical application. Therefore, further research is necessary to identify tumor-related biomarkers that can predict the prognosis of immunotherapy. Tertiary lymphoid structures (TLSs) have been recognized as a crucial factor in predicting the response of immune checkpoint inhibitors in solid tumors, including CM. However, the study of TLSs in CM is not yet comprehensive. Gene expression profiles have been shown to correlate with CM risk stratification and patient outcomes. In this study, we identified TLS-related genes that can be used for prognostic purposes and developed a corresponding risk model. The impact of TLS-related genes on clinicopathological characteristics, immune infiltration and drug susceptibility was also explored. Our biological function enrichment analysis provided preliminary evidence of related signaling pathways. Our findings provide a new perspective on risk stratification and individualized precision therapy for CM.

## Introduction

Cutaneous melanoma (CM) is one of the most lethal types of skin cancer with increasing prevalence and mortality^1^. CM accounts for high proportion of skin cancer deaths by its high rate of invasion and distant metastasis^1^. Immune checkpoint blockade (ICB) has attracted much attention in recent years due to its remarkable efficacy in the CM clinical application^2^. Compared to surgical treatment in the early phase of the disease, the efficacy of immune checkpoint blockade remains limited despite the effective response of immune checkpoint blockade in a wide range of cancer types, and not limited by the status of specific gene mutations^3^. PD-L1 expression is by far the most widely used predictive marker approved to guide immunotherapy. However, various anti-PD-L1 /PD-1 agents that have been used in clinical practice have different PD-L1 detection antibodies/kits and interpretation criteria/thresholds. Some patients of PD-L1 negative can also benefit from immunotherapy, while some patients PD-L1 positive do not respond to treatment. The inconsistency between PD-L1 expression and immunotherapy poses a challenge to clinical application^2,4^. Therefore, the imperfect nature of this marker should be recognized and tumor related biomarkers related to the prognosis of immunotherapy should be continuously investigated.

With the deepening of human research on cancer, it is clear that the prognosis of tumor is tightly linked to its stage, grade, malignant degree and other factors. Among the above factors, tumor immune microenvironment (TME) catches increasing attention, which is greatly correlated with the development, recurrence, metastasis and drug resistance of tumors^5^. Tumor infiltrating lymphocytes (TILs) in TME has been demonstrated can reflect the prognosis and disease progression of tumor patients^6^. Tertiary lymphoid structures (TLS) are aggregates of TILs stimulated by chronic inflammation in tumors^7,8^. As reviewed in 2022, TLS cell composition, location, density, maturation status and other related factors have been proved to be useful in evaluating the prognosis of multiple tumors, including melanoma^8^. Studies have shown that for most tumor types, TLS invasion in TME is beneficial for tumor prognosis. The higher the TLS density, the higher the degree of infiltration and the higher the maturity, the better the clinical prognosis of the patient^9^. However, due to the existence of tumor heterogeneity, the level of immune cell infiltration in different types of tumors is also different, and the prognostic value of TLS is also different^10^. No significant correlation between TLS and prognosis has been reported in both neuroendocrine tumors and hepatocellular carcinoma^11,12^. Breast cancer peritumoral TLS was even identified as a factor for poor prognosis^13^. Therefore, it is necessary to further elucidate the prognostic value of TLS in tumors.

Increasing evidence indicates that TLS can served as a predictor of immune checkpoint inhibitor (ICI) efficacy, which is independent from PD-L1 expression^14^. Given the success of ICI in melanoma, the significance of tumor-associated TLS for response to immunotherapy was investigated in CM patients. An RNA-seq study based on cancer tissue from 177 melanoma patients demonstrated that different stages of TLS exist in different stages of CM^15^. The investigators collected melanoma biopsies that had received CTLA4 blockade and grouped them based on the molecular characteristics of TLS. Survival analysis data indicated that, for patients treated by CTLA4 blockade or PD-1-related therapy, higher TLS expression levels was significantly related to better prognosis than lower TLS expression^14,15^. Although TLS signature molecules have a good prognostic effect, studies in CM are not comprehensive, and relevant prognostic models is still lacking for further prognostic risk stratification of CM and individualized treatment guidance.

A new risk model comprising of three TLS-associated genes (TLS-RGs) has been developed, and its prognostic significance has been successfully verified. Differences related to risk stratification in clinicopathological characteristics, immune infiltration landscape, and drug sensitivity have been studied, which could provide a new perspective for individualized and precise treatment. Additionally, functional enrichment analysis has provided preliminary evidence for the signal pathways involved in this risk model.

## Materials and methods

### Dataset downloads of cutaneous melanoma patients

We retrieved the gene expression matrix and clinical data of cutaneous melanoma (CM) patients from the TCGA and GEO databases, respectively. In addition, we collected transcriptome data of normal tissues from CM patients using the UCSC Xena database. To ensure the reliability of our study, we excluded CM patients who lacked overall survival (OS) value or had a survival time of less than 0 days. Ultimately, our study included 454 CM samples from TCGA and 210 from GSE65904. Perl scripts were utilized to extract the transcriptome matrix of CM from TCGA and GEO, and the transcriptome matrix symbol was annotated by the ensembles human genome browser GRCh38.p13. While excluding M stage due to the remarkable difference in CM sample size, we included other clinicopathological features of CM patients such as survival time, survival status, age, gender, stage, and T/N stage for further analysis.

### Consensus clustering analysis

We utilized the "ConsensusClusterPlus" package in R software to cluster the CM samples into different molecular subtypes based on the prognostic three TLS-RGs. We determined the number of clusters using the K-means method, and the analysis was repeated 1000 times to ensure the stability of the classification.

### Risk model construction of prognostic TLS-related genes

A total of 39 tertiary lymphoid structures-related genes (TLS-RGs) were identified from the previous research to construct the risk model for CM^15^ (Supplementary Table 1). Univariate Cox regression analysis and subsequent LASSO algorithm were used to screen prognostic TLS-RGs. We subsequently carried out multivariate Cox regression analysis to further identify the TLS-RGs related to the CM patients’ prognosis and then constructed a novel risk model. We calculated the risk score of each sample based on the following formula: = (-0.244 × CCL8) + (-0.121 × CXCL13) + (0.187 × IRF4). Then, the CM samples were randomly sub-categorized into the low- and high-risk groups on the basis of the median threshold.

### Validation of TLS-RGs risk model

The TCGA database obtained CM samples were randomly stratified into the training cohort (n = 318) and the test cohort (n = 136) in a 7:3 ratio. According to the median risk score value in the training cohort, we clustered CM samples in the training and test cohorts into high- and low-risk groups, respectively. Besides, GSE65904 was used as an external validation cohort to verify the independence and stability of the risk signature.

### Construction and validation of nomogram

To assess the independence of the risk model, we performed univariate and multivariate Cox regression analysis by R package “survival”. The R package “pROC” was used to evaluate the reliability of TLS-RGs risk mode. The R package “timeROC” was carried out to plot the ROC curve of the 1-, 3-, and 5-year for OS rates of CM. Then, we developed a nomogram that incorporated risk score and several clinicopathologic features by R package “rms”. The concordance index (C-index) plot, decision curve analysis (DCA) and calibration diagram were carried out to evaluate the predictive ability of the nomogram from multiple perspectives.

### Tumor immune analysis and drug sensitivity analysis

According to ESTIMATE algorithm, we calculated the stromal score, immune score and ESTIMATE score, and tumor purity by R package “estimate”. The CIBERSORT and a single sample gene set enrichment analysis (ssGSEA) algorithm were performed to explore the immune infiltration landscape of CM. The result of immunophenoscore (IPS) was evaluated from the TCIA database (https://tcia.at/home). Moreover, we acquired the expression level of immune checkpoint inhibitor (ICI) from the TCGA matrix using the R package “limma”. We retrieved the tumor immune dysfunction and exclusion (TIDE) scoring file from the the TIDE database (http://tide.dfci.harvard.edu). Besides, IMvigor210 cohort (an immunotherapy cohort) was utilized to predict the reactions of anti-PD1/PD-L1. According to the TLS-RGs risk model, the risk score in the IMvigor210 cohort was calculated to evaluate the response of patients in the SD/PD and CR/PR group. Additionally, we explore potential relationship between drug sensitivity (IC50) and TLS-RGs risk model by the R package “pRRophetic”.

### Cell culture and qRT-PCR analysis

The A375 and HFB4 cell lines were obtained from ATCC. To initiate culture, the frozen stock of A375 and HFB4 cells were thawed in a 37 °C water bath and transferred to sterile 15 mL centrifuge tubes containing 10 mL of DMEM/F12 culture medium. Subsequently, the tubes were incubated in a humidified incubator at 37 °C with 5% CO2. RNA extraction from both cell lines was performed using Trizol reagent (Cat# 15596018, Thermo), followed by cDNA synthesis using an RT kit with gDNA Eraser (Perfect Real Time). Real-time quantitative qRT-PCR (Cat# RR047A, Takara) was carried out for further analysis. Finally, the mRNA expression levels were measured using SYBR Pre-mix Ex Taq II (TliRNaseH Plus) (Cat# RR820B, Takara).

### Tumor mutation burden analysis

After obtaining tumor mutation data of the CM patients from TCGA databases, we utilized perl scripts to extract the mutation data from the raw data. A waterfall plot was utilized by the “Maftools” package.

### Functional enrichment analysis

Differentially expressed genes (DEGs) between high-and low-risk groups were identified based on the criteria of |Fold Change|≥ 2, and p < 0.05. Then we applied gene ontology (GO) and Kyoto Encyclopedia of Genes and Genomes (KEGG) analysis to complete functional enrichment analysis of DEGs by “clusterProfiler” package^16^.

### Statistical analysis

Statistical analyses were performed using R (version 4.1.0) and Perl scripts. Spearman-ranked correlation analysis was conducted to examine the correlation between two gaps, and a *p*-value of < 0.05 was considered statistically significant. Differential functions between the two groups were analyzed using the Wilcoxon rank-sum test, with statistical significance set at a *p*-value of < 0.05.

## Results

### Identification of prognostic TLS-RGs

**Figure 1:**
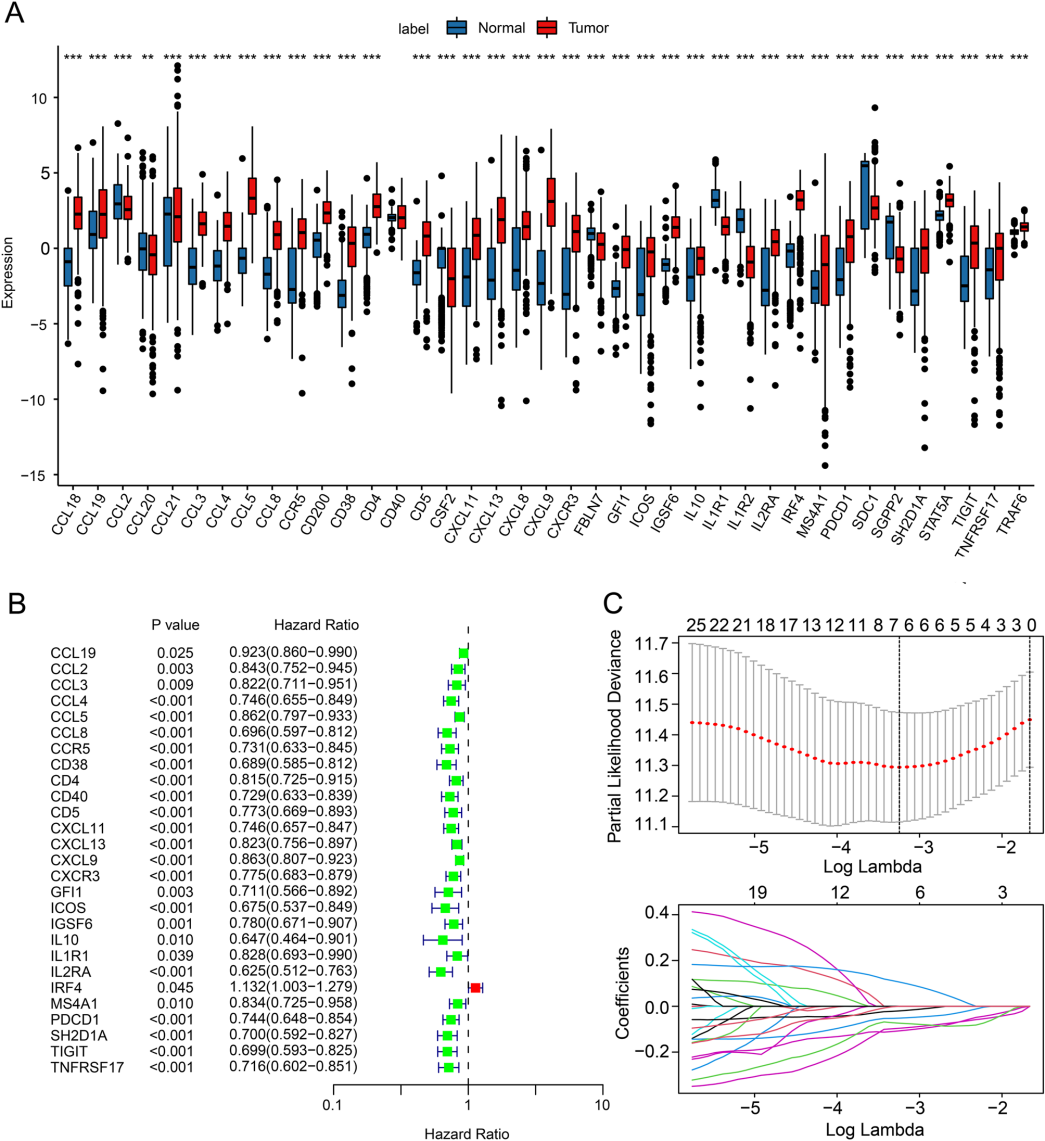
Risk model development based on the TLS-RGs signature. (**A**) Expression of TLS-RGs in CM patients and paired normal samples. (**B**) Univariate Cox regression analysis of prognostic TLS-RGs. (**C**) LASSO regression analysis of prognostic TLS-RGs.

A total of 39 TLS-RGs were collected for next study. The expression level of TLS-RGs was significantly different in tumor tissues compared to that in the corresponding normal tissues (Fig. 1A). According to the univariate Cox regression analysis result, 7 prognostic TLS-RGs were obtained via the LASSO analysis (Fig. 1B,C). Finally, 3 prognostic TLS-RGs (CCL8, CXCL13 and IRF4) were identified for the subsequent analysis, which could be regarded as independent prognostic predictors of CM determined by multivariate Cox regression analysis.

### Consensus clustering for TLS-RGs associated with prognosis and immune infiltration landscape in CM

**Figure 2:**
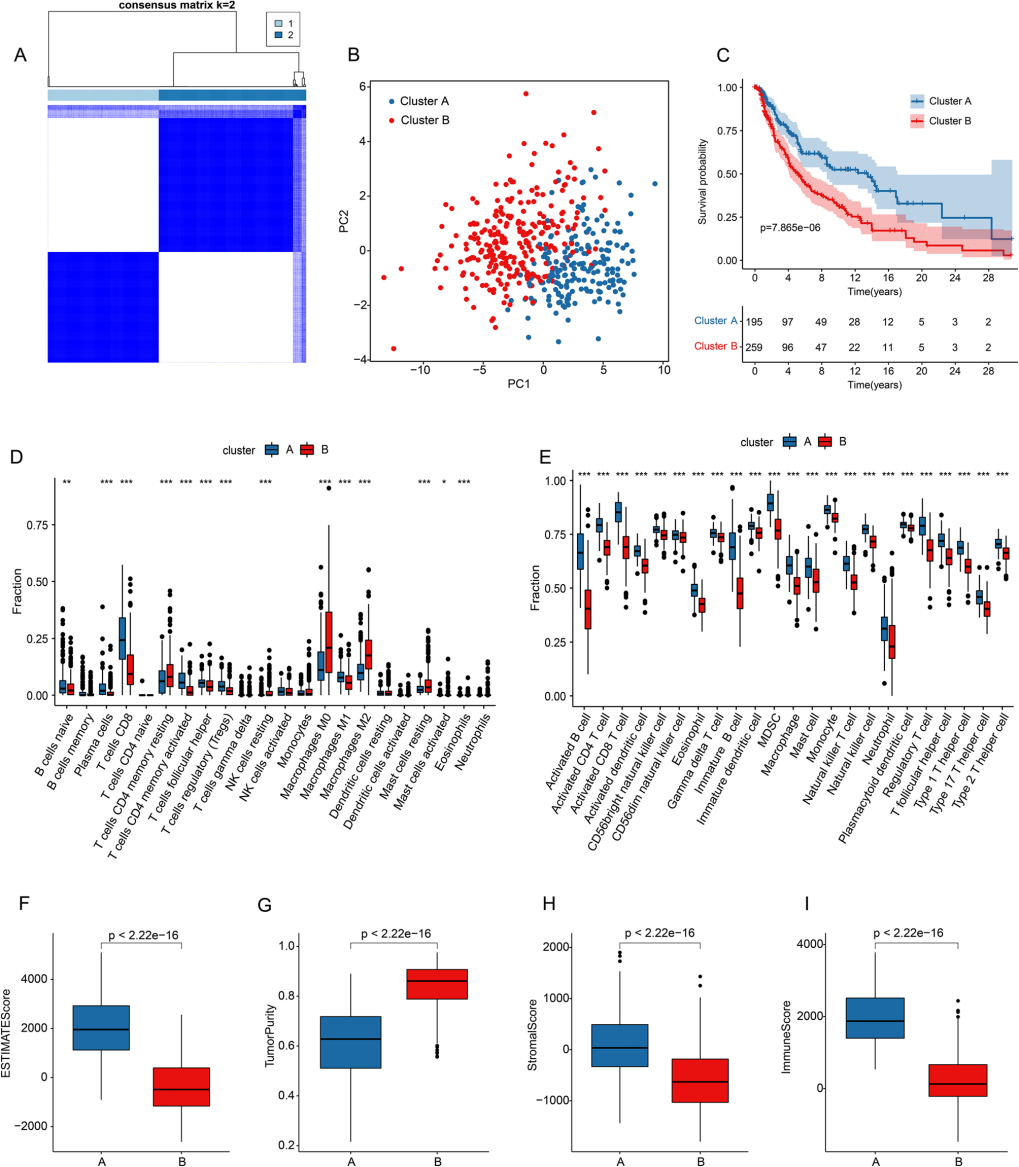
Consensus clustering and immune infiltration analysis in CM. (**A**) Molecular subtyping by consensus clustering heatmap. (**B**) Principal component analysis of CM patients between Cluster A and B. (**C**) Kaplan–Meier survival analysis between Cluster A and B. Difference in fractions of immune cells between the two subtypes via (**D**) CIBERSORT and (**E**) ssGSEA algorithm. Characteristic analysis between the two clusters by (**F**) ESTIMATE score. (**G**) Tumor purity. (**H**) Stromal score. (**I**) Immune score.

The molecular subtypes of CM were identified according to the TLS-RGs risk model in CM, and the CM samples were clustered into Cluster A (n = 195) and Cluster B (n = 295) by consensus clustering analysis. (Fig. 2A). Principal component analysis (PCA) plot suggested that the CM samples in Cluster A and B could be clearly distinguished (Fig. 2B). The Kaplan–Meier survival analysis showed the OS rate of patients in Cluster A was greatly higher than that in Cluster B, indicating a clear difference in clinical outcome of CM (Fig. 2C). The CIBERSORT and ssGSEA algorithm were utilized to quantify the proportions of immune cells of CM patients. The CIBERSORT result showed a significant difference in proportion of tumor-infiltrating immune cells between the two clusters (Fig. 2D). The ssGSEA result suggested that 23-type immune cells were upregulated in Cluster A (Fig. 2E). In addition, higher ESTIMATE, stromal and immune scores as well as lower tumor purity were observed in Cluster A by ESTIMATE algorithm (Fig. 2F–I).

**Figure 3:**
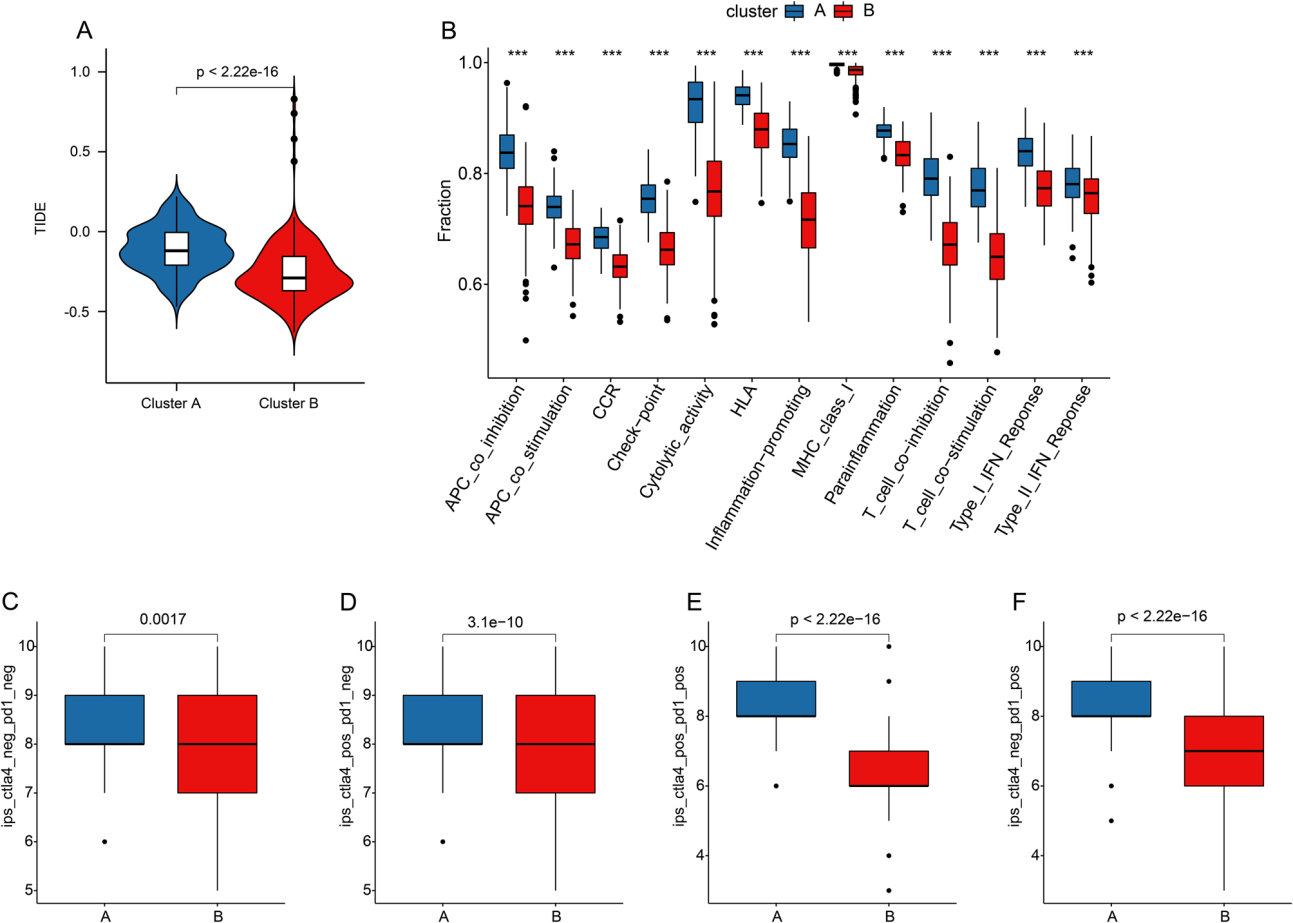
Association of TLS-RGs risk model and immunotherapy response in CM. (**A**) TIDE score analysis between the two clusters. (**B**) Difference in immune function score between the two subtypes Immune function score. (**C**–**F**) IPS score showed the possible response ability to immunotherapy of CM patients in Cluster A and Cluster B.

The difference in immunotherapy efficiency in two molecular subtypes was further evaluated. Tumor immune dysfunction and exclusion (TIDE) result showed lower TIDE score in Cluster B, suggesting a potential better benefit to immunotherapy (Fig. 3A). Immune function analysis illustrated a higher immune function score of CM patients in Cluster A as compared with that in Cluster B (Fig. 3B). Immunophenoscore (IPS) results showed a clear difference in immunotherapy response of CM samples between Cluster A and Cluster B, indicating CM samples in Cluster A showed a better response to PD-1, and PD-1/CTLA4 treatment (Fig. 3C–F).

### Validation of TLS-RGs risk model

**Figure 4:**
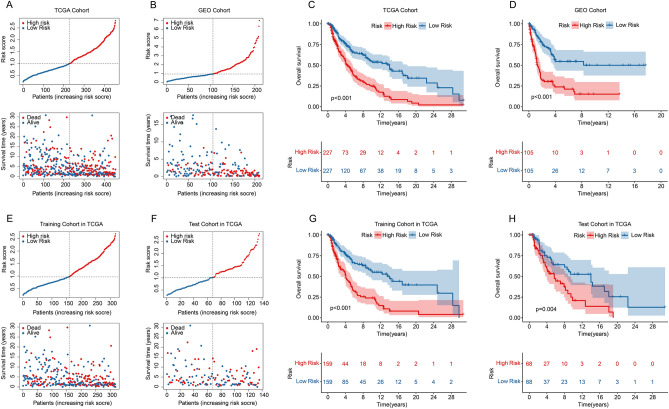
Validation of TLS-RGs risk model in CM. The distribution of risk score and the scatter dot plot shows the correlation of survival time and TLS-RGs prognostic signature in (**A**) TCGA, (**B**) GEO, (**E**) Training, and (**F**) Test cohorts. (**B**) Kaplan–Meier survival curve showed OS differences between the low- and high-risk groups in (**C**) TCGA, (**D**) GEO, (**G**) Training and (**H**) Test cohorts.

**Figure 5:**
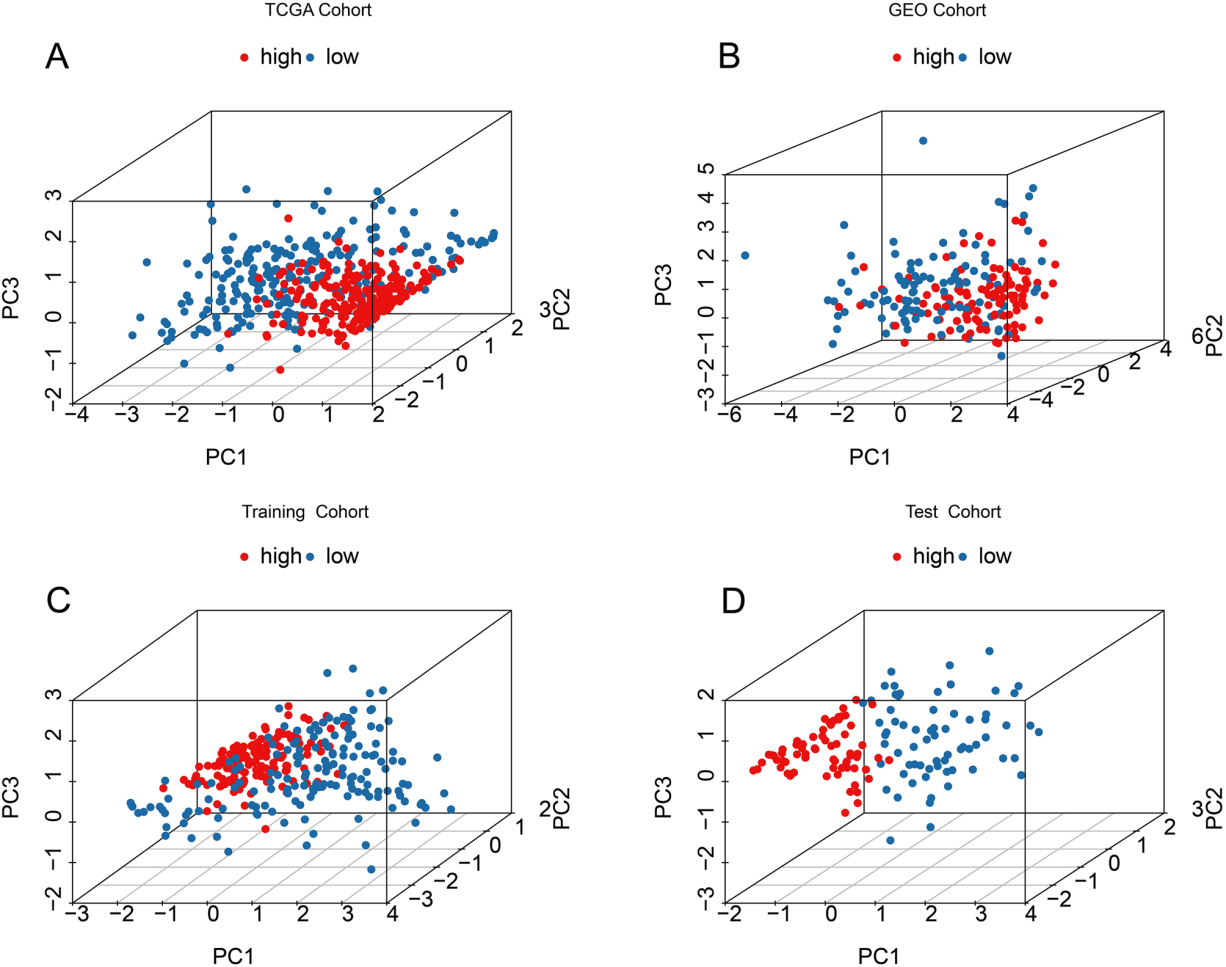
PCA analysis of TLS-RGs risk model. 3D PCA plots showed significant distributions by risk stratification based on TLS-RGs risk model in (**A**) TCGA, (**B**) GEO, (**C**) Training and (**D**) Test cohorts.

The CM patients were categorized into the high-risk group and low-risk group based on the median risk score. The scatter dot plot indicated higher mortality risk in CM patients with increasing risk scores (Fig. 4A). Kaplan–Meier survival analysis revealed that high risk score was significantly associated with poor prognosis of CM patients (Fig. 4C). An internal and external validation cohort were subsequently developed to validate the prognostic significance of TLS-RGs risk model. The CM patients in training, test and GEO cohorts, were segregated into high-and low-risk group based on the median risk score, and the scatter dot plot revealed an inverse association of TLS-RGs prognostic signature and survival time (Fig. 4B,E,F). The Kaplan–Meier survival curve analysis suggested that the patients with high-risk scores were associated with worse OS rate in training, test and GEO cohorts (Fig. 4D,G,H). PCA score plots indicated that prognostic signature had acceptable ability to distinguish well by risk stratification in TCGA, training, test and GEO cohorts (Fig. 5A–D). These results demonstrated that the risk model could accurately predict the prognosis of patients with CM.

### Independent prognosis analysis of risk model

**Figure 6:**
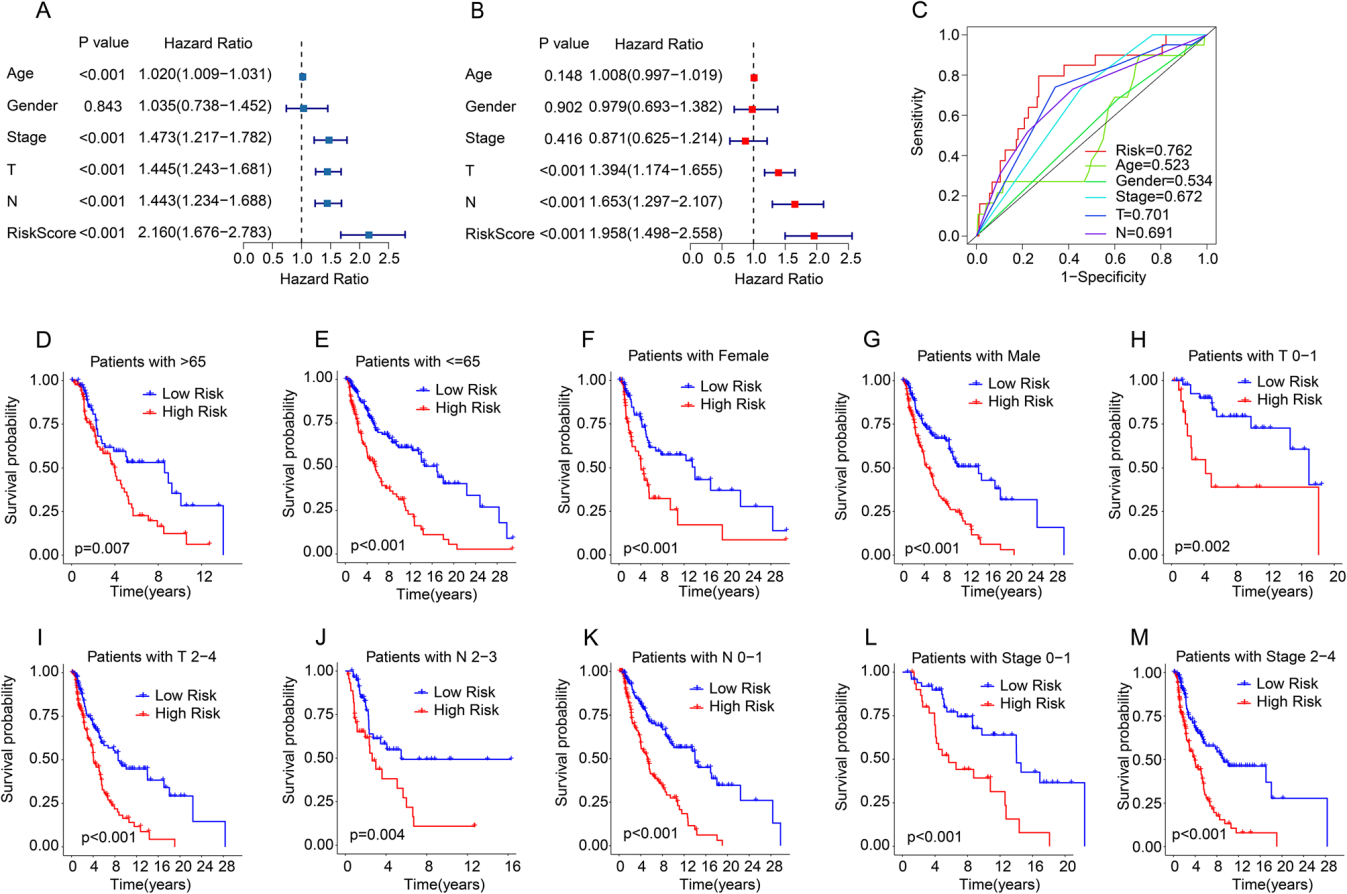
Independent prognosis analysis based on TLS-RGs risk model and clinicopathological characteristics. (**A**,**B**) Univariate and multivariate Cox regression analysis of the novel risk model. (**C**) ROC curve of TLS-RGs risk model and clinicopathology characteristics. (**D**–**M**) Kaplan–Meier survival curve of CM patients with low- and high-risk scores in different clinicopathological characteristics.

The univariate and multivariate Cox analyses for risk score and other clinicopathologic feature were performed in the training cohort. The univariate Cox regression analysis result suggested that age, stage, T, N, and risk score (all *p* < 0.001) were remarkably associated with clinical outcome of CM patients (Fig. 6A). Then, T, N, and risk score (all *p* < 0.001) were determined as independent prognostic predicting factors for CM by multivariate Cox regression analysis (Fig. 6B). The ROC curve suggested the risk model had higher predictive ability than other clinicopathological characteristics (Fig. 6C). Moreover, the prognostic value of TLS-RGs prognostic signature in different clinicopathological features was further investigated using stratified subgroup analysis. CM patients were separated into low- and high-risk group in the different clinicopathological characteristics, involving in age (age > 65 vs age ≤ 65), gender (female vs male), T (T 0–1 vs T 2–4), N (N 0–1 vs N 2–3), stage (stage 0–1 vs stage 2–3). The survival analysis suggested that compared to high-risk group, the OS rate of CM patients was much higher in the low-risk group in the different clinicopathological characteristics (Fig. 6D–M). These results demonstrated that the prognostic signature could independently predict the prognosis of CM patients relative to different clinicopathological variables.

### Construction and validation of Nomogram

**Figure 7:**
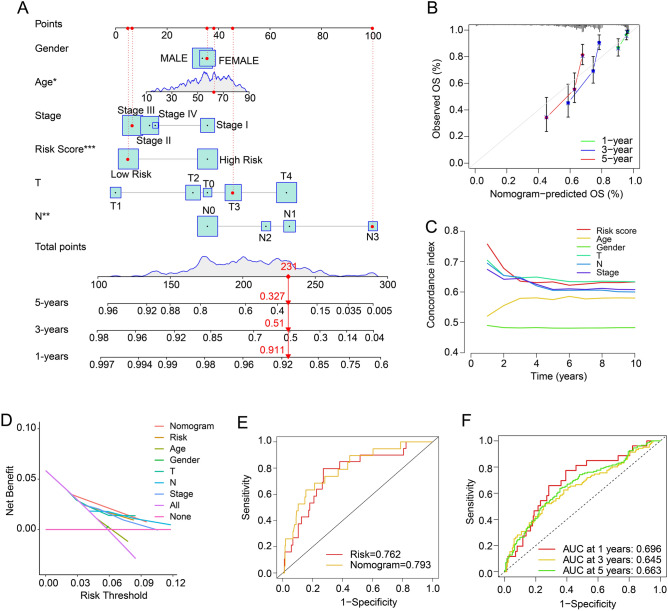
Nomogram model based on TLS-RGs risk score and clinicopathological characteristics for CM patients. (**A**) A hybrid nomogram model to evaluate survival probability of CM patients based on TLS-RGs prognostic signature and clinicopathological characteristics. (**B**) Calibration curve to test the stability of nomogram result. (**C**) Concordance index (C-index) and (**D**) Decision curve analysis (DCA) for the nomogram. (**E**) ROC curve of TLS-RGs risk model and nomogram. (**F**) Time-dependent ROC curve.

According to TLS-RGs prognostic risk model and clinicopathological features, we established a fresh nomogram for CM patients 1-, 3-, and 5-year OS predicting (Fig. 7A). The calibration curve showed the consistency of predicted and actual OS, suggesting the perfect stability of the nomogram (Fig. 7B). Concordance index (C-index) suggested that risk score had higher C-index compared to other clinicopathological characteristics (Fig. 7C). Decision curve analysis (DCA) revealed a satisfactory predictive capacity of nomogram in evaluating survival probability (Fig. 7D). The AUC of nomogram was 0.793, which was higher than risk score (Fig. 7E). Time- dependent ROC curve analysis suggested that the AUC was 0.696, 0.645 and 0.663 for the 1-, 3-, and 5-years ROC, respectively (Fig. 7F). These results indicated that the nomogram is reliable and precise in predicting survival probability of CM patients.

### Immune infiltration landscape analysis

**Figure 8:**
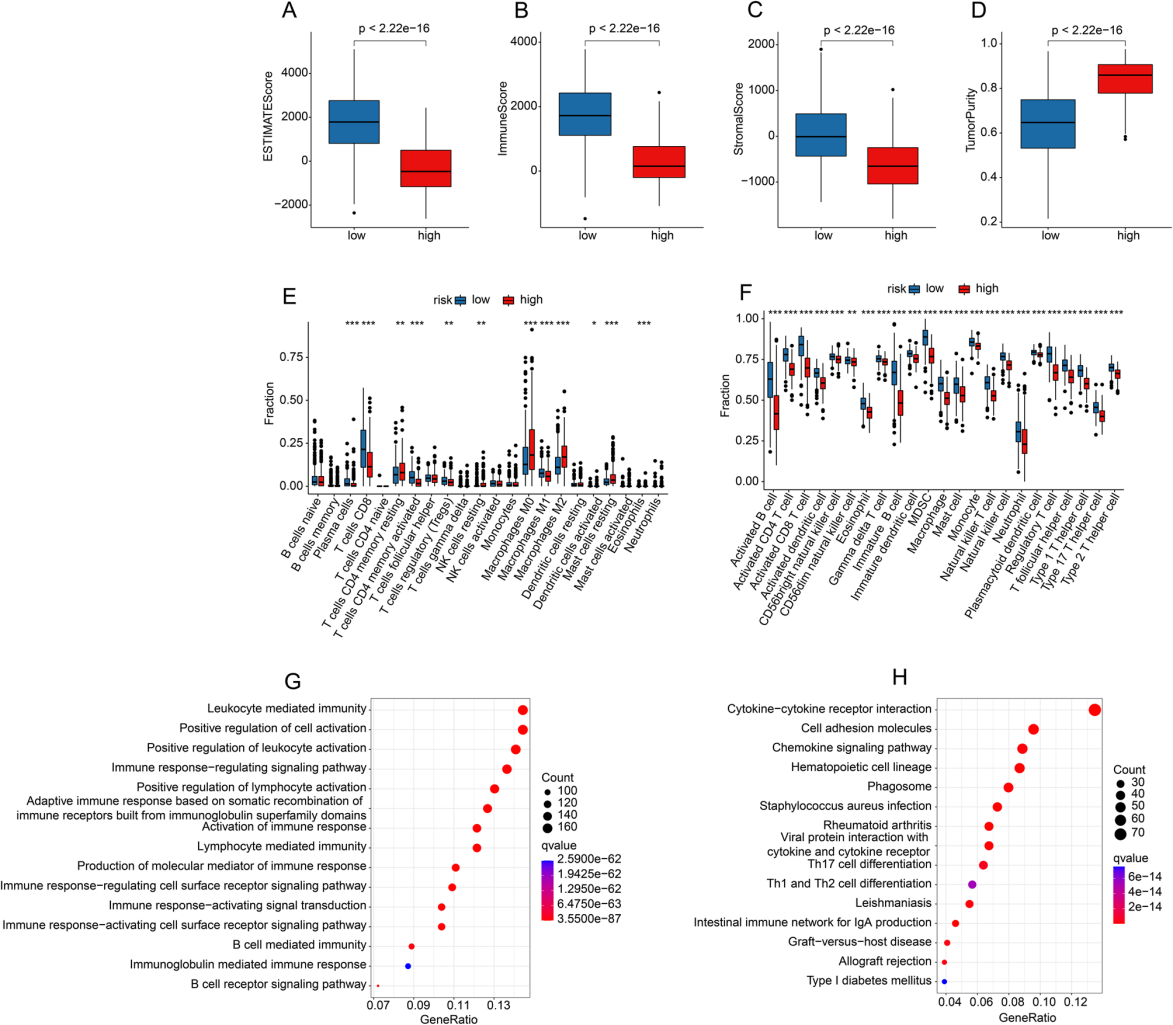
Immune infiltration and enrichment analysis in low- and high-risk groups. (**A**) ESTIMATE score, (**B**) immune score, (**C**) stromal score, and (**D**) tumor purity between the low- and high-risk groups. The proportions of immune cells by risk stratification were calculated by (**E**) CIBERSORT and (**F**) ssGSEA algorithms, respectively. (G) GO and (H) KEGG enrichment analysis of DEGs in low- and high-risk groups, the DEGs were set at |Fold Change|≥ 1 and P < 0.05. (Data sources: www.kegg.jp).

We performed multiple immune assessment algorithms to investigate the correlation between the risk score and the tumor immune microenvironment in CM patients. In terms of TME scores, lower tumor purity, as well as higher immune, stromal, and ESTIMATE scores were observed in the low-risk group via ESTIMATE algorithm (Fig. 8A–D). Additionally, the CIBERSORT result indicated significant difference in proportion of tumor-infiltrating immune cells between high-and low-risk groups (Fig. 8E). The ssGSEA result indicated that patients with high-risk score had higher proportion of 23-type immune cells than those in low-risk group, indicating an immunosuppressed status of patients in the high-risk group (Fig. 8F).

To investigate the molecular functions and biological processes that potentially associated with the established TLS-RGs signature, we employed the enrichment analysis to analyze the differentially expressed genes (DEGs) by risk stratification. The GO result indicated that the DEGs were significantly associated with cellular component (CC) and molecular function (MF), involving in a series of immune related processes (Fig. 8G, Supplementary Table 2). Furthermore, several pathways involved in tumor immunity were also highlighted through KEGG analysis and included cytokine − cytokine receptor interaction (Fig. 8H). These results demonstrated that immune-associated biological functions may be significantly involved in the role of TLS-RGs in tumorigenesis of CM.

### The immunotherapy response and drug sensitivity analysis

**Figure 9:**
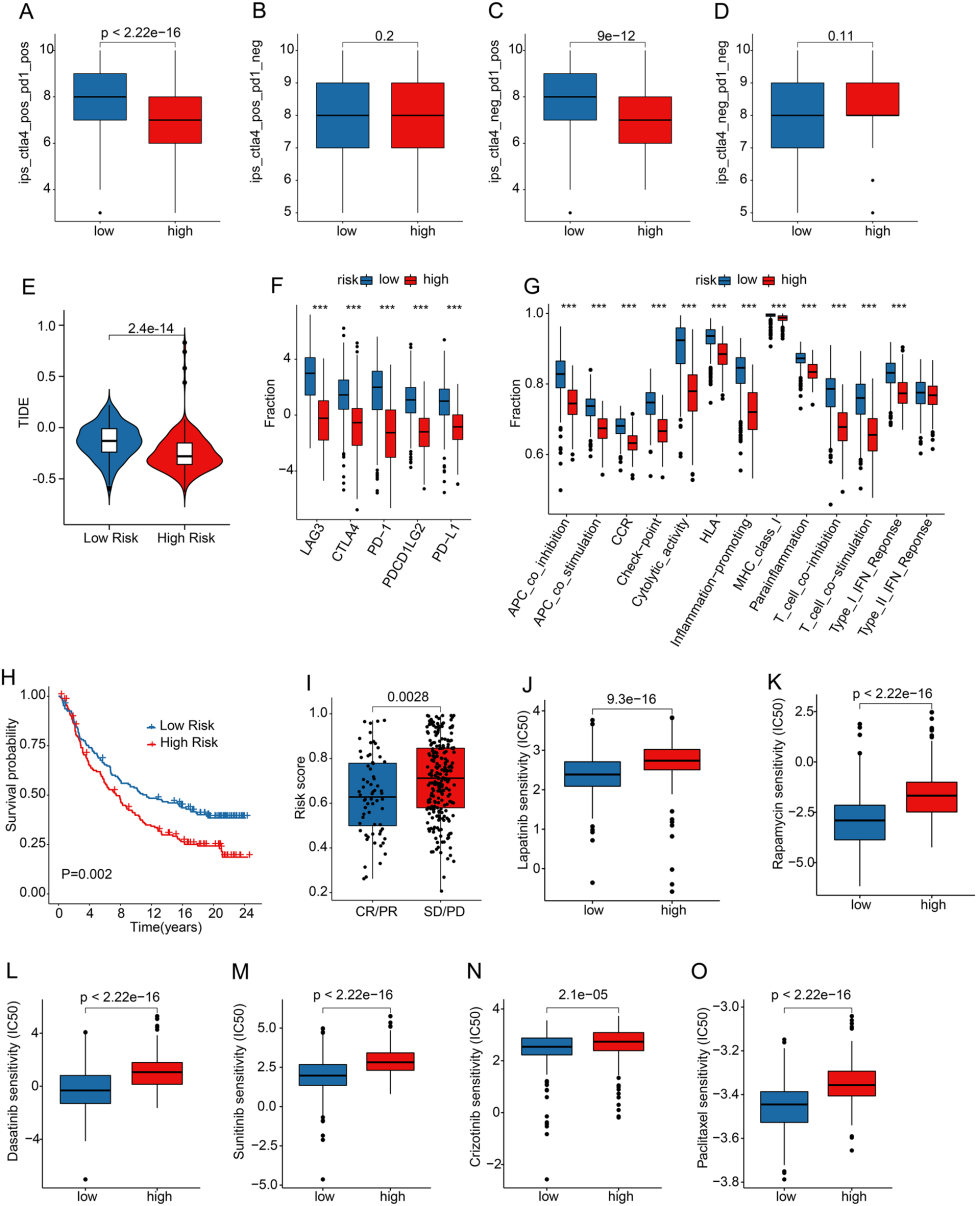
Immunotherapy response and drug sensitivity analysis. (**A**–**D**) IPS score and (**E**) TIDE score between the low- and high-risk groups. (**F**) The expression of immune checkpoint inhibitors (ICIs) by risk stratification. The expression of ICIs is transformed by log 2 (expression + 1). (**G**). Immune function score of CM patients between different risk groups. (**H**) The Kaplan–Meier survival curve of patients with low- and high-risk scores in the IMvigor210 cohort. (**I**) The risk score of patients in CR/PR and SD/PD groups in the IMvigor210 cohort. PR, Partial Response, PD, Progressive Disease, SD, Stable Disease, and CR, Complete Response. Drug sensitivity (IC50) of (**J**) Lapatinib, (**K**) Rapamycin, (**L**) Dasatinib, (**M**) Sunitinib, (**N**) Crizotinib, and (**O**) Paclitaxel.

The responses to immunotherapy of CM patients were further evaluated. IPS showed patients in low-risk cohort had a more promising response to the PD-1, CTLA-4, and PD-1/CTLA-4 treatment (Fig. 9A–D). Moreover, TIDE was applied to demonstrate the predictive power of risk scores for immunotherapy. The TIDE score was negatively related to the risk score, suggesting better immunotherapeutic efficacy for patients in high-risk cohort (Fig. 9E). Differences of immune checkpoint inhibitor expression were also compared in the high- and low-risk groups, and *LAG3, CTLA4, PD-1, PDCD1LG2,* and *PD-L1* were significantly upregulated in the low-risk group (Fig. 9F). Immune function result suggested drastic differences between high-and low-risk group, such as CCR, cytolytic activity, and T cell co − inhibition were expressed at higher levels in the low-risk group (Fig. 9G). Meanwhile, we further investigate the role of risk score in the efficacy of immunotherapy using IMvigor210 (an anti-PD-1/PD-L1 treatment cohort). Kaplan–Meier survival curve revealed a relatively poor clinical outcome for CM patients with high-risk score (Fig. 9H). Besides, the risk score of patients in CR/PR was significantly lower than in SD/PD in the IMvigor210 cohort (Fig. 9I). Above results demonstrated that the risk score is associated with immunotherapy response, which could be utilized as a predictor of immunotherapy response for CM patients.

Targeted drug therapy and chemotherapy were also very important critical strategy for CM. Thus, which forced us to subsequently investigate the drug sensitivity (IC50) of several potential antineoplastic drugs. As shown in Fig. 9J–O, higher IC50 of lapatinib, rapamycin, dasatinib, sunitinib, crizotinib, and paclitaxel was observed in the low-risk patients, suggesting that risk score could evaluate drug sensitivity, and might provide a new perspective for individualized treatment of CM patients in the future.

### Tumor mutation burden landscape analysis

**Figure 10:**
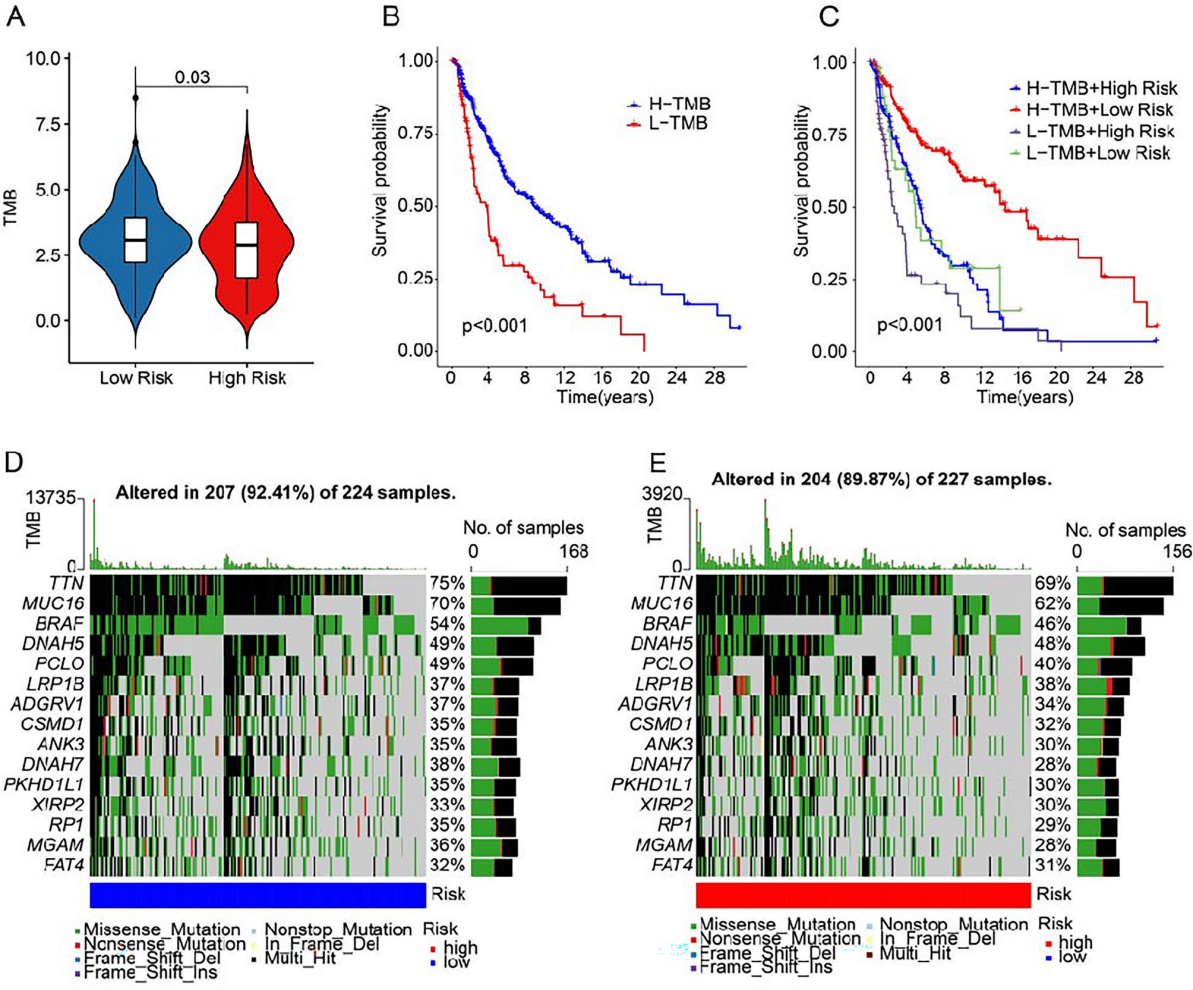
Tumor mutation burden landscape of CM patient in the low- and high-risk group. (**A**) TMB analysis. (**B**) The Kaplan–Meier survival curve of CM patients with low- or high-TMB. (**C**) The Kaplan–Meier survival curve of CM patients in H-TMB + high-risk, H-TMB + low-risk, L-TMB + high-risk, and L-TMB + low-risk groups. (**D**,**E**) Waterfall plots of the top 15 genes in the mutant landscape.

Tumor mutation burden (TMB) was considered as a novel biomarker predictive of tumor response to cancer immunotherapy^17^. Patients in low-risk group had higher TMB level compared to the other group (Fig. 10A). The Kaplan–Meier survival analysis showed differences in OS between the high-TMB and low-TMB groups (Fig. 10B). Patients with higher TMB were correlated to better clinical outcome. Besides, we found patients in the low-risk + H-TMB group had the highest OS rate than other groups (Fig. 10C). The waterfall plots showed highest gene mutation frequencies of in both groups, such as *TTN, MUC16, BRAF, DNAH5,* and *PCLD* (Fig. 10D,E).

### Validation of TLS-RGs in the clinical samples

**Figure 11:**
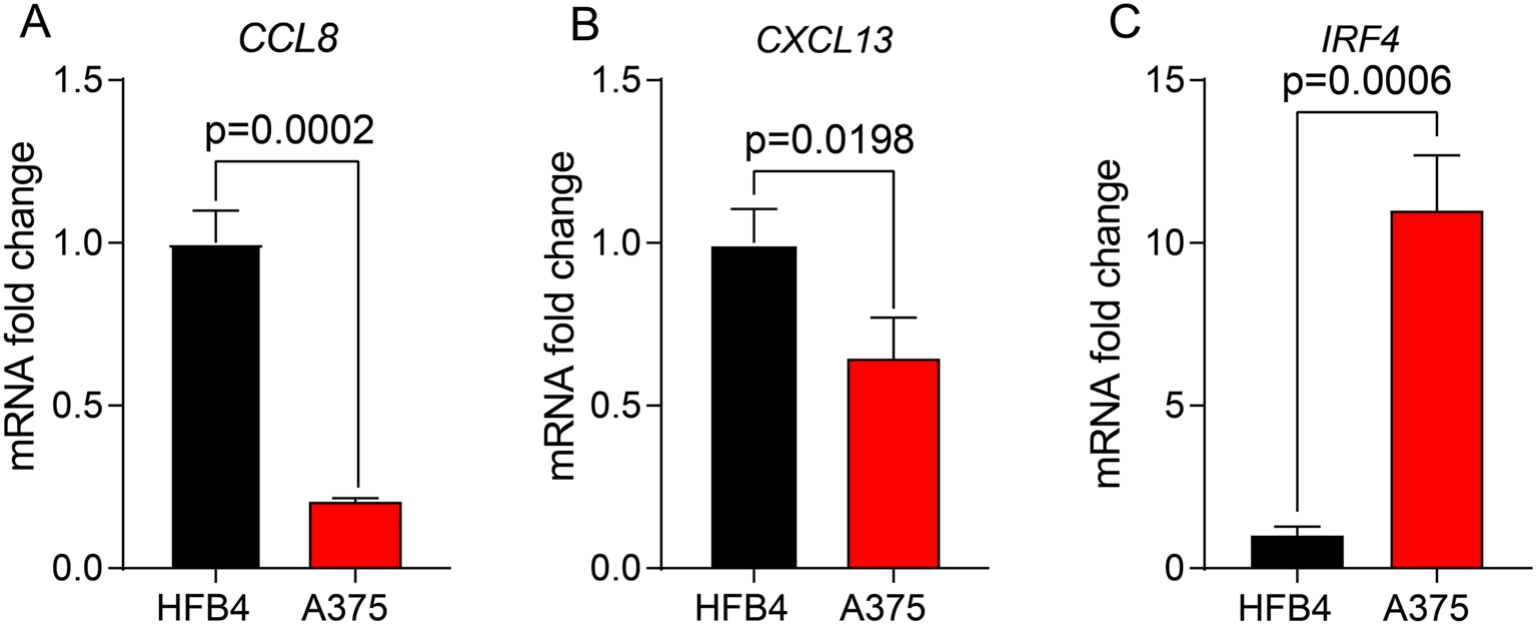
The mRNA expression level of 3 TLS prognostic signature. The mRNA level of (**A**) CCL8; (**B**) CXCL13; (**C**) IRF4 in HFB4 and A375 cell lines.

We utilized the qRT-PCR method to explore CCL8, CXCL13 and IRF4 expression level in melanoma A375 cell lines and HFB4 control cell line. The results revealed that CCL8 and CXCL13 expression were downregulated in A375 cell line, while IRF4 was upregulated in HFB4 cell line, which was consisted with the TCGA and GEO cohorts (Fig. 11).

## Discussion

CM is one of the most aggressive malignancies, leading to a significant proportion of tumor-related deaths. Based on three TLS related genes, we first constructed a risk model and validated its ability to predict prognosis in CM patients. We preliminarily explored the possible mechanisms involved in this process subsequently. As a result, the relationship between this prognostic model and the immune microenvironment was explored as a possible reason for predicting CM outcomes. Additionally, the different expressions of immune checkpoint markers and chemo agent sensitivities provide a new perspective on clinical treatment options.

A strong association was demonstrated between TLS and immune microenvironment of melanoma^16^. In the TLS related risk model we established, 3 markers with prognostic value were selected out. Higher IRF4 expression level was observed in the patients with high-risk score than those group had a relatively. IRF4 is an important transcription factor for Treg cell differentiation in TME and is associated with the formation of tumor suppressor microenvironment^18^. In addition, in melanoma patients, IRF4 up-regulates the transcriptional level of lncRNA TEX41 in melanoma cells, which inactivates miR-103a-3p and its downstream C1QB, thereby promoting the proliferation, migration and invasion of melanoma cells and inhibiting cell apoptosis^19^ It has also been reported that the IRF4-IRF1 axis influences the effect of immunotherapy by regulating melanoma immunogenicity^20^. Our data also showed a negative correlation between IRF4 expression level and a variety of immune cell components. Our data show that CCL8 expression is decreased in the CM patients with high-risk score, and CCL8 can trigger chemotactic activity of monocytes, lymphocytes, basophils, and eosinophils^21^. The expression of CCL8 plays an important role in tumorigenesis of a variety of tumors^22,23^. Pretreatment levels of CCL8 were previously reported to be consistently elevated in CM patients who did not respond to immune checkpoint inhibitors compared with patients who responded to ICI^24^. Higher CCL8 expression was observed in baseline melanoma biopsies from patients with ICI resistance^25^. However, CCL8 has been reported to exert the protective effect in CM^26^. Our results support that CCL8 level correlates with a better prognosis of CM patients. At the same time, compared to high-risk group, the lower multidrug sensitivity was found in the low-risk group with higher CCL8 expression. Given the contradictory results, the role of CCL8 in CM response to ICI needs to be further analyzed with larger scale data.

As a chemoattractant, the role of CXCL13 in tumorigenesis has been reported. It was previously reported that CXCL13 recruits B cells to the TME and differentiates into regulatory B cells. These regulatory B cells play a significant role in the formation and regulation of immunosuppressive TME^27^. CXCL13 is also related to the process of epithelial mesenchymal transition (EMT) in tumor cells. Silencing CXCL13 is a potential anti-tumor tool^27^. However, CXCL13 has been reported to have a different effect in immunotherapy. CXCL13 is associated with better survival after immunotherapy in patients with advanced bladder cancer and can predict ICI response in advanced bladder cancer patients^28^. Based on the widespread use of immunotherapy in CM, our evidence in CM also supports that CXCL13 is prognostic factor and predicted better outcome after immunotherapy.

In our TLS-related prognostic model, TMB level was higher in the low-risk group and associated with a higher OS rate. This is consistent with literature reports that TMB is positively correlated with prognosis in cutaneous melanoma^29^. Melanoma has an ideal response to immunotherapy because of its immunogenicity^30^. Previous clinical trials have shown that among melanoma patients, those with high TMB may benefit more from immune checkpoint related therapy^31^. This may explain why high TMB is correlated with better clinical outcome. TTN, MUC16, and BRAF were most frequently mutated genes in descending order. As an oncogenic driver in melanoma, BRAF mutation can be used as a target for precision therapy of CM^32,33^. Mutations in TTN are commonly detected and are associated with increased TMB and a better response to ICI in solid tumors. Therefore, mutated TTN can be used as a marker for better prognosis of patients^34^. MUC16 mutations have been reported to be involved in the increased expression level of immune checkpoint in melanoma patients^35^. We get the same trend. In addition, more DC were activated in the MUC16 mutant sample^36^. Combined with evidence that DC promotes immune activation in the TME and is significantly related to immune responses^37,38^, this could also explain why MUC16 mutations are associated with betters OS rate.

In conclusion, a risk model consisting of three TLS-related genes was constructed in our study, which is independent and reliable in predicting prognosis of CM. In addition, our study can not only further guide the clinical risk stratification of CM, the results on immune microenvironment, immune response and drug sensitivity analysis provides new perspectives and insights for individualized and precise treatment strategies for CM patients.

### Supplementary Information

Supplementary Tables.

## Data Availability

The datasets generated and/or analysed during the current study are available in the repositories of GEO and TCGA. The dataset identifier for GEO is GSE65904.
