# Supplementary material for: The molecular subtypes and clinical prognosis characteristic of tertiary lymphoid structures-related gene of cutaneous melanoma
Source: Sci Rep. 2023 Dec 28;13:23097. doi: 10.1038/s41598-023-50327-6 (PMC10754817; doi:10.1038/s41598-023-50327-6)
Supplement: Supplementary file 1 — Supplementary Tables. [file 41598_2023_50327_MOESM1_ESM.docx]

Supplementary Material

**Supplementary Table 1** Tertiary lymphoid structure related genes

| No. | Gene | No. | Gene | No. | Gene |
| --- | --- | --- | --- | --- | --- |
| 1 | CCL18 | 14 | CD40 | 27 | IL1R1 |
| 2 | CCL19 | 15 | CD5 | 28 | IL1R2 |
| 3 | CCL2 | 16 | CSF2 | 29 | IL2RA |
| 4 | CCL20 | 17 | CXCL11 | 30 | IRF4 |
| 5 | CCL21 | 18 | CXCL13 | 31 | MS4A1 |
| 6 | CCL3 | 19 | CXCL8 | 32 | PDCD1 |
| 7 | CCL4 | 20 | CXCL9 | 33 | SDC1 |
| 8 | CCL5 | 21 | CXCR3 | 34 | SGPP2 |
| 9 | CCL8 | 22 | FBLN7 | 35 | SH2D1A |
| 10 | CCR5 | 23 | GFI1 | 36 | STAT5A |
| 11 | CD200 | 24 | ICOS | 37 | TIGIT |
| 12 | CD38 | 25 | IGSF6 | 38 | TNFRSF17 |
| 13 | CD4 | 26 | IL10 | 39 | TRAF6 |

**Supplementary Table 2** Immunological function related results in GO enrichment analysis

| ID | Description | GeneRatio | qvalue | Count |
| --- | --- | --- | --- | --- |
| GO:0002443 | leukocyte mediated immunity | 164/1136 | 5.88E-84 | 164 |
| GO:0050867 | positive regulation of cell activation | 164/1136 | 3.55E-87 | 164 |
| GO:0002696 | positive regulation of leukocyte activation | 160/1136 | 2.39E-85 | 160 |
| GO:0002764 | immune response-regulating signaling pathway | 155/1136 | 7.03688E-71 | 155 |
| GO:0051251 | positive regulation of lymphocyte activation | 148/1136 | 4.46305E-82 | 148 |
| GO:0002460 | adaptive immune response based on somatic recombination of immune receptors built from immunoglobulin superfamily domains | 144/1136 | 4.59337E-79 | 144 |
| GO:0002449 | lymphocyte mediated immunity | 138/1136 | 6.24353E-74 | 138 |
| GO:0002440 | production of molecular mediator of immune response | 126/1136 | 1.3623E-69 | 126 |
| GO:0002429 | immune response-activating cell surface receptor signaling pathway | 118/1136 | 1.26225E-64 | 118 |
| GO:0002757 | immune response-activating signal transduction | 118/1136 | 1.26225E-64 | 118 |
| GO:0002429 | immune response-activating cell surface receptor signaling pathway | 118/1136 | 1.26225E-64 | 118 |
| GO:0019724 | B cell mediated immunity | 101/1136 | 6.79255E-64 | 101 |
| GO:0016064 | immunoglobulin mediated immune response | 99/1136 | 2.58625E-62 | 99 |
| GO:0050853 | B cell receptor signaling pathway | 82/1136 | 4.72398E-64 | 82 |
